# Structures and Functional Diversities of ASFV Proteins

**DOI:** 10.3390/v13112124

**Published:** 2021-10-21

**Authors:** Guoguo Wang, Mengjia Xie, Wei Wu, Zhongzhou Chen

**Affiliations:** State Key Laboratory of Agrobiotechnology and Beijing Advanced Innovation Center for Food Nutrition and Human Health, College of Biological Sciences, China Agricultural University, Beijing 100193, China; SZ20193020193@cau.edu.cn (G.W.); sz20203020189@cau.edu.cn (M.X.); wuweiyou@cau.edu.cn (W.W.)

**Keywords:** African swine fever virus, ASFV, structures, survival, AP endonuclease, dUTPases, pS273R, p72, *Asfv*PolX, *Asfv*LIG

## Abstract

African swine fever virus (ASFV), the causative pathogen of the recent ASF epidemic, is a highly contagious double-stranded DNA virus. Its genome is in the range of 170~193 kbp and encodes 68 structural proteins and over 100 non-structural proteins. Its high pathogenicity strains cause nearly 100% mortality in swine. Consisting of four layers of protein shells and an inner genome, its structure is obviously more complicated than many other viruses, and its multi-layered structures play different kinds of roles in ASFV replication and survival. Each layer possesses many proteins, but very few of the proteins have been investigated at a structural level. Here, we concluded all the ASFV proteins whose structures were unveiled, and explained their functions from the view of structures. Those structures include ASFV AP endonuclease, dUTPases (E165R), pS273R protease, core shell proteins p15 and p35, non-structural proteins pA151R, pNP868R (RNA guanylyltransferase), major capsid protein p72 (gene *B646L*), Bcl-2-like protein A179L, histone-like protein pA104R, sulfhydryl oxidase pB119L, polymerase X and ligase. These novel structural features, diverse functions, and complex molecular mechanisms promote ASFV to escape the host immune system easily and make this large virus difficult to control.

## 1. Introduction

African swine fever (ASF) is an acute hemorrhagic viral disease caused by the African swine fever virus (ASFV), affecting wild boar and domestic pigs. ASFV is the only member of the *Asfarviridae* family which is an enveloped icosahedral deoxyvirus and shares similar structural, genomic, and replicative characteristics with other nucleocytoplasmic large DNA viruses (NCLDVs) [1]. A great deal of large DNA viruses have been found in the past two decades, and few of them have caused as great an economic impact to society as ASFV [2]. ASFV has a contagious nature and easily spread features that make it one of the most terrible diseases [3]. An area epidemic causes nearly 100% mortality in swine, and all pigs in the area will be slaughtered in order to control the virus, which leads to huge economic losses. However, there are no commercial vaccines or medicines at present [4].

ASFV is a linear double-stranded DNA virus whose genome ranges from 170 to 193 kbp [5]. It contains 151~167 open reading frames (ORFs) that are closely arranged and read from both DNA strands. The differences in gene number and genome length of ASFV variations are mainly due to gains or losses of ORFs from the multigene families (MGF). The genome termini of ASFV are covalently closed by imperfectly base-paired hairpins, resembling that of poxvirus genomes [6]. The genes were usually named based on gene orientation left or right (L or R) and number of residues. Moreover, the promoter sequences of each gene are generally short and AT rich.

**Figure 1:**
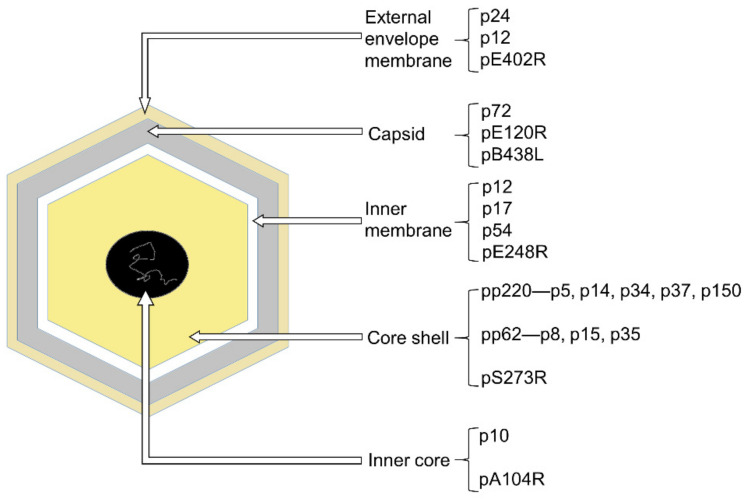
Illustration of ASFV particle and examples of proteins in each layer. The ASFV is composed of five layers: the external envelope membrane (pale-yellow), the capsid (gray), the inner membrane (white), the core shell (yellow), and the inner core (black).

The ASFV particle is an icosahedral multi-layered structure, possessing bunch of polypeptides whose characteristics are largely unknown [5]. This large and complex DNA virus containing five layers [7,8] (Figure 1). The outermost layer is the external envelope membrane, which is similar to the plasma membrane in accordance with the budding process [9]. No matter the existence of this external envelope membrane, ASFV is infectious. Thus, the existence of this external envelope membrane not only protects this large virus, but also complicates the invasion and budding process. Beneath the external envelope membrane is the layer of the capsid. The structure of the capsid has been studied in detail, revealing about 2800 capsomers with the appearance of hexagonal prisms. Additionally, capsomers within a trisymmetron all pack in essentially the same orientation, rotating by ~60° from the capsomers in neighboring trisymmetrons and creating 30 zippers (cleavage lines) on the capsid [7,8]. Additionally, it has a maximum diameter of 250 nm. Under the capsid is the inner membrane which shows a 70Å thick single lipid bilayer structure by TEM and is derived from the endoplasmic reticulum [10,11]. The remaining layers consist of the core shell and the inner core. The core shell is defined as an independent domain of the virus [12], 180 nm diameter, and mainly constituted by polyprotein 220 (pp220) and polyprotein 62 (pp62), both of which can be cleaved by protease pS273R. In that way, pp62 can form p8, p15 and p35, and pp220 turns into p5, p14, p34, p37 and p150. Additionally, the inner core is a genome-containing nucleoid layer surrounded by the thick protein layer (core shell) [7], with a histone-like protein pA104R. As we can see, ASFV has multiple and intricate layers, and each layer contains different kinds of proteins (Figure 1). Besides those structural proteins in-layers, there are a large quantity of non-structural proteins. Some of them participate in the evasion of host defenses, including type I interferon and cell death pathways [13]. The vast majority of ASFV proteins are still waiting to be explored.

ASFV mainly targets monocyte/macrophages and dendritic cells [14], the natural hosts of ASFV include wild suids and arthropod vectors of the *Ornithodoros* genus [4]. Wild boars are also sensitive to ASFV, showing similar symptoms or subclinical symptoms to domestic pigs, and can directly transmit the virus to domestic pigs. ASFV infection is asymptomatic in the natural reservoir hosts such as ticks [15]. There is a tick and domestic pig circulation that ticks feed on the blood of infected pigs with ASFV, carrying and spreading ASFV to pigs. Therefore, protection in domestic pigs is isolate specific [16]. More importantly, the viruses can survive for up to 8 years in the ticks, ticks also carry ASFV throughout their whole life, and the virus can spread between ticks through mating [17,18]. The virus is large and most virus–host interactions remain to be elucidated. Moreover, immunity to ASFV is complex and the host receptors remain unknown. Therefore, understanding the structures, the key residues, and the molecular mechanisms of this large and complex virus becomes greatly important to developing a vaccine.

## 2. A Few Structures, Functions, and Mechanisms of ASFV Proteins Have Been Revealed

ASFV possesses a large genome that varies from 170 kbp to 193 kbp and encodes over 150 proteins. Although the whole cryo-EM structure of ASFV was reported [7,8], their resolution was not enough to resolve the structural detail of each protein component. To date, only a few structures of ASFV have been revealed, some of which are structural proteins, while some are expressed to perform special functions for viral survival. Here, we summarized structural features, functions, and molecular mechanisms of structure-unveiled proteins.

### 2.1. AsfvAP, AsfvPolX, and AsfvLIG Are Essential in ASFV Genome Repair and Variation

ASFV is replicated and assembled in the swine macrophage cell with an oxidative environment [19]. In this oxidative environment, free oxygen radicals are very rich and cause continuous damage to the ASFV genome [19], such as producing DNA mutations, abasic sites, DNA break, base modifications, strand crosslink, and protein-DNA adducts [19]. To overcome these damages efficiently, ASFV developed its own base excision repair (BER) system, including an aurinic/apyrimidinic endonuclease (*Asfv*AP), a ligase (*Asfv*LIG), and a repair polymerase (*Asfv*PolX). Greatly different from other repair enzymes, the fidelities of both *Asfv*PolX and *Asfv*LIG are very low partly due to their smallest protein size [20,21]. Thus, these enzymes play important roles in maintaining the viral genome and forming ASFV variants and genotypes, which further complicate the epidemiology and prevention of ASFV.

**Figure 2:**
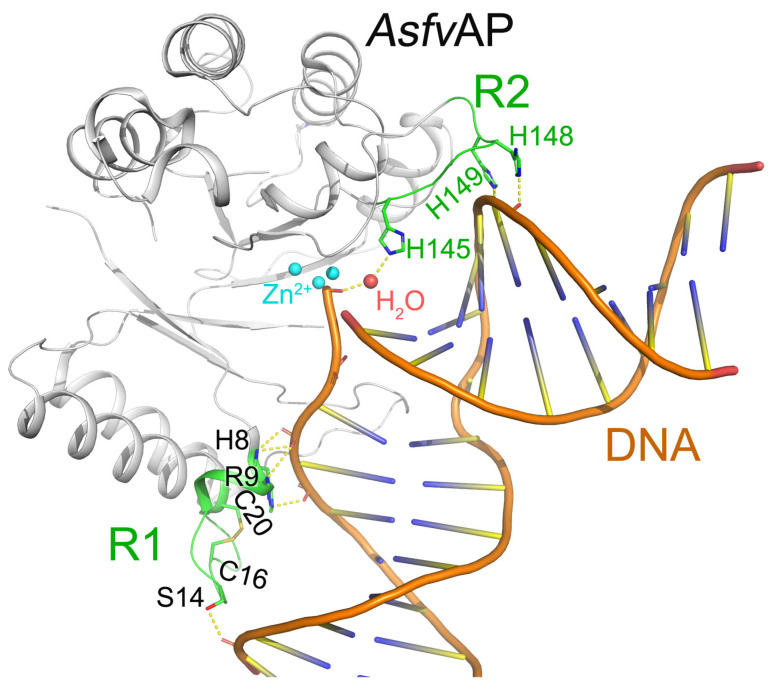
Detailed interactions between DNA and *Asfv*AP (pdb: 6KI3) [19]. Regions R1 and R2 are colored green. The interacting residues and the disulfide bond Cys16-Cys20 of R1 are highlighted by sticks.

*Asfv*AP participates in the BER pathway of ASFV and is essential for ASFV survival in the host cells [22]. Differed from other Aps, *Asfv*AP adopts a unique DNA-binding mode [19]. Additionally, it also has new structural features, including one narrower nucleotide-binding pocket at the active site, a histidine-rich region R2, and a C16–C20 disulfide bond containing region R1 (Figure 2). These features are important for *Asfv*AP to adapt to the acidic and oxidative environment [23]. The residues outside the nucleotide-binding pocket are important in assembling *Asfv*AP and in binding nucleotide. For example, the disulfide bond formed between Cys16 and Cys20 is important for *Asfv*AP function. Although the S-S bond does not interact with DNA directly, the region R1 containing disulfide bond plays important roles in binding DNA (Figure 2). When the reducing reagents such as DTT break the S-S bond, the conformation of R1 will be changed and the *Asfv*AP’s DNA binding ability is compromised [19]. The *Asfv*AP structure also has a histidine-rich R2 loop that directly interacts with the pre-cleaved DNA strand. Moreover, the R2 loop can interact with the 5′-phosphate group of 5′-deoxyribose phosphate through one water molecule (Figure 2) [19]. However, *Asfv*AP prefers acidulous condition to exert its AP endonuclease activity. When the environment pH is higher than 6.3, *Asfv*AP’s activity will be reduced.

To date, ASFV DNA ligase (*Asfv*LIG) is found to be one of the most error-prone ligases, catalyzing the DNA repair process of ASFV. Ligation or further elongation induces a two-unit concatemer with dimeric ends that may generate the genomic DNA by site-specific nicking, rearrangement [24]. Therefore, it seems that the error-prone mechanism is beneficial to the ASFV replication by contributing to quick genotype formation [25], which will arise the livability and the variability of ASFV. *Asfv*LIG is one of the smallest DNA ligases identified at the moment. Interestingly, *Asfv*LIG serves a substrate-binding function by using the unique N-terminal domain (NTD) that has no similarity to DNA-binding domains in any other ligases. Besides the NTD, *Asfv*LIG contains other two domains, one is the adenylation domain (AD) in the center and the other is the OB-fold domain (OB) in the C-terminus. AD and OB domains are both conserved in homologous proteins such as human DNA ligases [26,27].

ASFV DNA polymerase X (*Asfv*PolX) is one of the smallest known nucleotide polymerase [20] and lacks proofreading 3′-5′ exonuclease, resulting in the lower fidelities of polymerase X than other polymerases [23,28,29]. A great conformational adjustment of helix αE was observed in NMR concentration of perturbation [20]. It creates a larger space for a bulky base pair or positions the templating base, so it would be partially complementary to a ‘mismatched’ nucleotide. Therefore, *Asfv*PolX can play a crucial role during the rapid mutations of the viral genome. As compared with the conserved homologous proteins, it lacks two DNA-binding domains (the thumb domain and 8–KD domain) [21]. Moreover, *Asfv*PolX has higher catalytic efficiencies when filling in gapped substrates and when having a phosphate group at the 5′-margin of the gap [29]. Additionally, DNA binding is the rate-limiting step of the catalytic efficiency of *Asfv*PolX [30]. In a word, these three enzymes are important in the BER system, which repairs damaged DNA throughout the cell cycle, and are beneficial to the virus survival and evolution.

### 2.2. ASFV dUTPase and Guanylyltransferase

**Figure 3:**
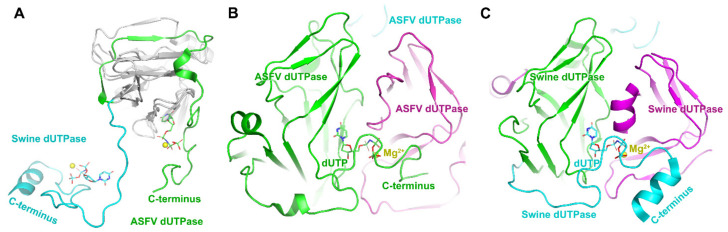
Structures of ASFV dUTPase and swine dUTPase. (**A**) Subunit superimposition of ASFV dUTPase (pdb: 6LJ3) and swine dUTPase (pdb: 6LJJ) [31]. (**B**) The novel two-subunit active sites in ASFV dUTPase. (**C**) The classical three-subunit active sites in swine dUTPase. Different subunits are indicated by different colors. The C-terminus is highlighted. The dUTP is indicated as sticks and colored by element. The magnesium ion is indicated by the yellow sphere.

ASFV contains a gene encoding dUTPase (*E165R*) which is essential for viral replication, thus making it a spot for designing inhibitors. The dUTPase is closely related to the virulence and high-level replication efficiency of ASFV [31]. In addition, dUTPase only from ASFV is detected in quiescent macrophages. Thus ASFV dUTPase may be a good drug target. The host swine dUTPase, as a classical dUTPase, also possesses the classical trimeric dUTPase interactions: (i) extended bimolecular interfaces, (ii) three-fold central channels, and (iii) C-terminal β-strand swapping [32,33] (Figure 3). Comparing its structure with the swine enzyme, the ASFV dUTPase employs a novel two-subunit active site, but not three-subunit active sites in the classical dUTPase [31] (Figure 3). There are dual functions discovered in the ASFV dUTPase. One is catalyzing the hydrolysis of dUTP into dUMP and pyrophosphate, while the other is contributing to thymidylate biosynthesis when producing a precursor of dTTP. Therefore, dUTPase deletion from ASFV could directly affect viral replication in macrophages [34], and this nonclassical dual-subunit active center of ASFV dUTPase could be served as a potential antiviral target [31]. Selective inhibitors could be designed based on the significant volume difference of substrate binding pockets, different hydrophobicity distributions, and electrostatic distributions of the active sites between ASFV dUTPase and host dUTPase.

The ASFV protein pNP868R was found to have guanylyltransferase (GTase) activity involved in mRNA capping which is essential for mRNA stability and efficient translation of ASFV. The capping process usually requires three enzymatic reactions, including RNA 5′-triphosphatase, RNA guanylyltransferase, and RNA-methyltransferase [35,36]. In ASFV, the protein pNP868R was previously found to covalently bind to GTP (α-^32^P labeled), and pyrophosphate could reverse the complex, indicating that the GTase encoded by *NP868R* gene is involved in RNA capping [37]. It has a TPase domain, a GTase-OB fold domain, and an MTase domain. Structure-guided mutagenesis reveals potential vital residues involved in substrate binding, and many of them are conserved [38]. Both the role in MTase activities and the conformation of the N-terminal extension of ASFV pNP868R are distinct from those of other poxvirus MTase domain, suggesting a structural basis for potential anti-ASFV inhibitor design.

### 2.3. The p72 Is a Major Component of Capsid

The icosahedral capsid is mainly composed of 8280 copies of p72 and also has several minor capsid proteins [7,8]. According to previous experimental data, it is estimated that p72 accounts for about 32% of the total weight of virus particles. Every p72 contains two tandem roll domains, and each domain consists of 8 β-sheets. These domains are pretty common in the structure of the icosahedral virus capsid. The six roll barrels of the trimeric p72 together form a pseudohexamer with the bottom anchored to the inner membrane of the virus [7,39]. The inserts between the p72 folds of the trimer together form a propeller-like top structure that extends to the outside of the virus, most likely the receptor-binding area on the cell surface. The expression of p72 alone in HEK293F cells resulted in soluble aggregates. However, if co-expressed with B602L which is required for the formation of the icosahedral capsid and functions to aid the production of trypsin-resistant p72, p72 will be correctly folded [40]. The outer surface of p72 trimeric spike has plenty of charged residues. No obvious glycosylation site was observed in the Cryo-EM structure due to this high thermal stability of p72. At the same time, p72 is the main antigen detected in the blood of virus-infected pigs. The p72-based detection method is currently the most sensitive ASFV technology on the market.

**Figure 4:**
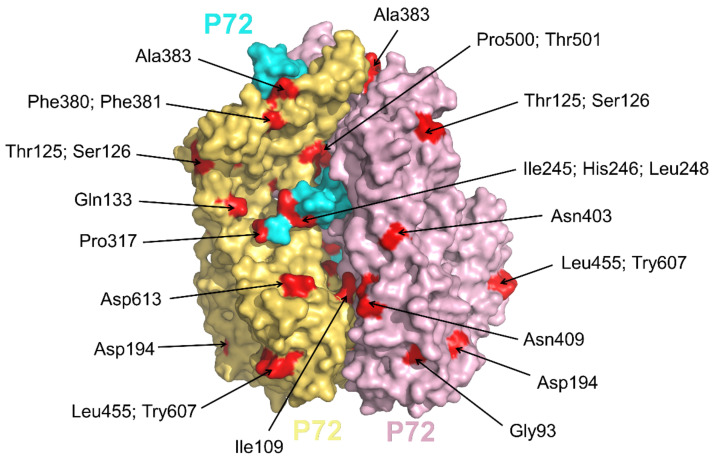
Location of mutations on the surface of p72 trimeric spike. Three subunits of p72 trimer (pdb: 6KU9) were shown in yellow-orange, cyan, and light-pink, respectively. All mutations of p72 were found by blasting all the full-length p72 at NCBI and were shown in red.

Based on the 3′-terminus of gene *B646L*, encoding the major protein p72, 24 different p72 genotypes were identified among the currently known virus isolates [41]. We analyzed the p72 C-terminus of the 24 genotypes mentioned above, mutations at P500, T501, I506, S526, V528, N569, A570, L586, V625, S626, and A627 were found and were labeled in Figure 4. Among these residues, P500 and T501 are almost covered by other p72 protein from the same p72 trimer, and only D613 (Figure 4) is located on the exposed top of the screw propeller-like cap [40]. Therefore, in the above 24 genotypes, the fact that few mutations on the epitopes were observed could not express the complexity of ASFV. One possible reason is that only 148 residues in C-terminus were reported in the above sequences and used for analysis. Then, we continued to search for all mutations of the full-length p72 at NCBI and the residues on the surface were shown in Figure 4. According to this analysis, the current approach to distinguish p72 genotypes may not represent the difference of the p72 epitopes and await further advance. 

### 2.4. Structural Proteins P15 and p35 Are Cleaved from pp62 by Protease pS273R

pp62, encoded by gene *CP530R*, is a polyprotein precursor of p8, p15, and p35. pp220, encoded by gene *CP2475L*, is a polyprotein precursor of p5, p14, p34, p37, and p150. pp62 and pp220 are cleaved by the specific SUMO-1 cysteine protease pS273R at the consensus sequence Gly-Gly-Xaa [42]. There are crosstalks between pp62 and pp220. pp62 processing requires the expression of pp220 [43], and the processing of both precursors pp62 and pp220 are dependent on the expression of p72. Moreover, pp62 has a profound impact on the subcellular localization of pp220, and both of them are located in the core shell [44].

ASFV p15 forms disulfide-linked trimers between the Cys9 and Cys30 from two different protomers [1]. The protomer secondary structural elements include four α-helix structures and six antiparallel β-strands. Additionally, two critical residues Lys10 and Lys39 are essential to the nucleic acid-binding affinity of ASFV p15. The disulfide bonds are important for the trimerization of ASFV p15, demonstrating a stronger binding affinity to ssDNA than to dsDNA [1].

The structure of ASFV p35 has a completely novel fold and is composed of two lobes of helix bundles, a larger “head domain” and a relatively smaller “tail domain” [45]. pH affects the oligomeric state of p35 in the solution. Dimers are observed at low pH and monomer is the main state of p35 in solution. These results imply that conformation change of p35 protein might happen during the virus entry and uncoating process. Moreover, p35 has one positive charged patch and a negatively charged one. Membrane association is mediated by a patch that contains a great deal of positive charges. Additionally, the patch containing negative charges can promote interaction between multiple structural proteins with other regions, further facilitating the binding of the core shell to the inner membrane [45].

ASFV pS273R is a SUMO-1 cysteine protease, catalyzing the maturation of the pp220 and pp62 polyprotein precursors into the core shell by cleavage. pS273R structure mainly consists of two domains named the “core domain” and the “arm domain” [46]. The “arm domain” contains the residues from M1 to N83, which shares no similarity to any structure fold, is unique to ASFV, playing an important role in the hydrolytic activity instead of stabilizing pS273R. Additionally, the “core domain” contains the residues from N84 to A273, sharing a high degree of structural similarity with chlamydial deubiquitinating enzyme [46].

pp220 and pp62, the main part of the core shell, are cleaved by the intrinsic pS273R protease to produce p5, p14, p34, p37, and p150 (derived from pp220) and p8, p15, and p35 (derived from pp62) [43,47]. This process is essential for ASFV particle maturation and infectivity. pS273R reduction inhibits polyprotein processing and profoundly impairs infective virus production. Thus, the pS273R protease is an attractive target for the design and development of effective inhibitors for ASFV treatments, similar to HIV and SARS-CoV2. The structural differences between ASFV pS273R and other proteases pave the way for the design of inhibitors to target this severe pathogen.

### 2.5. Sulfhydryl Oxidase pB119L and Thioredoxin pA151R

ASFV pB119L belongs to the Erv1p/Alrp family of sulfhydryl oxidases and has been described as a late nonstructural protein required for correct virus assembly, performing its activity by catalyzing the formation of disulfide bonds. It has the cysteines of its active-site motif CXXC [48]. Furthermore, pB119L has been suggested to interact with pA151R. Disulfide formation is essential for the proper assembly, maturation, propagation, and virulence of the viruses. The ASFV pB119L homodimerizes using an interface different from other sulfhydryl oxidases [49].

ASFV pA151R is a non-structural protein, which is expressed at both early and late stages of viral infection. ASFV pA151R was reported to associate with ASFV replication and assembly. Suppressing pA151R expression can reduce virus replication [50]. pA151R protein contains a WCTKC motif at its C terminus [51], which is similar to the thioredoxins active site motifs. However, the pA151R shares very low sequence identities with homologous proteins, leads to little understanding of its function and structural mechanisms [52,53]. The center of pA151R has a five-stranded beta-sheet, beside which are the two helices and the WCTKC motif. Notably, there are two cysteines in the WCTKC motif, and in particular, the thioredoxin active site-like feature WCTKC motif forms an architecture that is distinct from that in typical thioredoxins. Additionally, pA151R is found to form a monomer in solution [51].

### 2.6. pA104R, an Important DNA-Binding Protein in Replication

pA104R is a histone-like protein, and essential for ASFV genome packing and replication. It is located in the nucleoid and is related to nucleoid assembly [5]. pA104R binds to both single-stranded DNA (ssDNA) and double-stranded DNA (dsDNA), at the rate of ∼14–16 nt/bp, and displays DNA-supercoiling activity when coexists with ASFV topoisomerase II (pP1192R) [54]. The structure of pA104R is highly similar to other bacterial homologs with an α-helical “body”, a β-strand DNA binding region (BDR), and a flexible β-ribbon arm [55]. It is a homodimer formed by the α-helical “body” and the BDR. DNA duplex is simultaneously held by two pA104R dimers via the β-ribbon arms. The flexible β-ribbon arm binds DNA by contacting the major groove and is the crucial part of pA104R. When bound to pA104R, the DNA will be bent with an angle of 93.8°. Meanwhile, stilbene derivatives SD1 and SD4 disrupt the binding between pA104R and DNA, and thus inhibit the replication of ASFV in macrophages [55]. Due to the essential roles in ASFV nucleoid compaction and genome replication, pA104R is an important target for the development of ASFV chemotherapies.

### 2.7. A179L, a Potent Protein Inhibitor of Apoptosis

ASFV subverts apoptosis by encoding protein A179L, capable of sequestering proapoptotic host proteins and escaping the host immune system. A179L is an important inhibitor of apoptosis, it can stop HeLa cells and insect cells from apoptosis [56]. In addition to its role in apoptosis, A179L modulates autophagy via interacting with Beclin-1 and inhibits autophagosome formation under starvation conditions [57]. A179L adopts a Bcl-2 fold comprising 8 α-helices that form a globular helical bundle fold with a canonical ligand-binding groove [58]. The ability of A179L to bind proapoptotic Bcl-2 family members was systematically evaluated. It was found that A179L is the first panprodeath Bcl-2 protein binding to all major death-inducing mammalian Bcl-2 proteins including the BH3-only proteins. The BH3-only proteins are up-regulated in response to cellular damage, depriving growing factor, and activating the cell death pathway [59]. A179L can bind to many BH3-only proteins and block the cellular responses. The structures of complexes of A179L bound to Bid or Bax reveal the structural basis for apoptosis inhibition by ASFV [60]. A179L protein subverts Bcl-2-mediated apoptosis and increases the survival rate of ASFV by decreasing the autophagic activity and inhibiting the formation of autophagosome [60].

## 3. Conclusions

Structural information allows us to better understand the mechanisms of viral infection. In this review, we introduce the functions and structures of more than ten proteins, especially special domains or regions. For example, the structures and function of the ligase *Asfv*LIG and the replicase *Asfv*PolX reveal their error-prone nature. Thus, the mutation rate of ASFV is far higher than other regular DNA viruses. Due to the error-prone DNA repair mechanism, protein redundancy and rapid mutations, this large ASFV has always been a substantial challenge to design effective vaccines. Additionally, a structure-based vaccine design approach may improve the antigenicity and protective immune abilities. These structures explained how important they are in the ASFV lifecycle and the actual interactions at the molecular level.

The ASFV–host interactions play key roles in the ASFV replication and propagation. At moment, only a few ASFV–host structures of complexes are resolved. Therefore, there is still a huge space for viral proteins and host factors complex to provide structural bases and new insights for medical treatments against ASFV.

## Data Availability

Not applicable.
